# Supplementary material for: Development of Dispersion Process to Improve Quality of Hyaluronic Acid Filler Crosslinked with 1,4-Butanediol Diglycidyl Ether
Source: Polymers (Basel). 2024 Nov 27;16(23):3323. doi: 10.3390/polym16233323 (PMC11644390; doi:10.3390/polym16233323)
Supplement: Supplementary file 1 [file polymers-16-03323-s001.zip › polymers-3305530-supplementary.pdf]

Table S1. Complex viscosity of the filler material using dispersion process over time at 4℃ in lab scale(n=3).

Time(hr)	Complex viscosity(cP)	Standard deviation
0	2422431.53	1014882.61
1	2432039.81	349295.85
3	3097348.51	894504.65
24	2741333.66	662448.14

Table S2. Complex viscosity of the filler material using dispersion process over time at 10℃ in lab scale(n=3).

Time(hr)	Complex viscosity(cP)	Standard deviation
0	2422431.53	1014882.61
1	2852352.90	316606.66
3	3203046.67	480890.53
24	4798690.23	979996.24

Table S3. Complex viscosity of the filler material using dispersion process over time at 18℃ in lab scale(n=3).

Time(hr)	Complex viscosity(cP)	Standard deviation
0	2422431.53	1014882.61
1	2726067.34	399470.63
3	4266944.03	1287456.12
6	3569537.98	701382.61
17	4452989.22	381211.90
24	4307881.14	1051675.19

Table S4. Storage modulus (G′) of the filler material using dispersion process over time at 4℃ in lab scale(n=3).

Time(hr)	G′(Pa)	Standard deviation
0	305.61	122.12
1	304.23	43.65
3	386.95	111.31
24	342.96	82.73

Table S5. Storage modulus (G′) of the filler material using dispersion process over time at 10℃ in lab scale(n=3).

Time(hr)	G′(Pa)	Standard deviation
0	305.61	122.13
1	356.52	39.61
3	400.53	60.17
24	599.92	123.15

Table S6. Storage modulus (G′) of the filler material using dispersion process over time at 18℃ in lab scale(n=3).

Time(hr)	G′(Pa)	Standard deviation
0	305.61	122.13
1	339.29	39.61
3	532.28	160.54
6	445.09	86.55
17	554.75	47.68
24	537.87	130.83

Table S7. Loss modulus (G′′) of the filler material using dispersion process over time at 4℃ in lab scale(n=3).

Time(hr)	G′′(Pa)	Standard deviation
0	33.07	13.69
1	29.08	4.91
3	41.86	16.22
24	32.37	9.45

Table S8. Loss modulus (G′′) of the filler material using dispersion process over time at 10℃ in lab scale(n=3).

Time(hr)	G′′(Pa)	Standard deviation
0	33.07	13.69
1	37.04	3.77
3	39.58	7.94
24	60.77	7.62

Table S9. Loss modulus (G′′) of the filler material using dispersion process over time at 18℃ in lab scale(n=3).

Time(hr)	G′′(Pa)	Standard deviation
0	33.07	13.69
1	47.27	8.21
3	64.39	21.42
6	55.32	18.36
17	73.35	4.85

24	60.50	21.36

Table S10. Complex modulus(G*) of the filler material using dispersion process over time at 4℃ in lab scale(n=3).

Time(hr)	G* (Pa)	Standard deviation
0	307.39	122.89
1	305.61	43.93
3	389.21	112.49
24	344.48	83.27

Table S11. Complex modulus(G*) of the filler material using dispersion process over time at 10℃ in lab scale(n=3).

Time(hr)	G* (Pa)	Standard deviation
0	307.39	122.89
1	358.44	39.780
3	402.48	60.69
24	602.99	123.39

Table S12. Complex modulus(G*) of the filler material using dispersion process at over time at 18℃ in lab scale(n=3).

Time(hr)	G* (Pa)	Standard deviation
0	307.39	122.89
1	342.56	50.27
3	536.16	161.97
6	448.51	88.48
17	559.58	47.93
24	541.26	132.56

Table S13. Tack of the filler material using dispersion process over time at 4℃ in lab scale(n=3).

Time(hr)	Tack	Standard deviation
0	0.2420	0.0632
1	0.2486	0.0237
3	0.2605	0.0380
24	0.2831	0.0379

Table S14. Tack of the filler material using dispersion process over time at 10℃ in lab scale(n=3).

Time(hr)	Tack	Standard deviation
0	0.2420	0.0632
1	0.2517	0.0468

3	0.2874	0.0191
24	0.4065	0.0811

Table S15. Tack of the filler material using dispersion process over time at 18℃ in lab scale(n=3).

Time(hr)	Tack	Standard deviation
0	0.2420	0.0632
1	0.2315	0.0519
3	0.3134	0.0510
6	0.2808	0.0396
17	0.3475	0.0214
24	0.4251	0.0432

Table S16. Complex viscosity, storage modulus(G′) and loss modulus(G′′) of previous and new processes in the 1/2 factory scale (n=3).

Process	Complex viscosity(cP)	G′(Pa)	G′′(Pa)
No dispersion	2560882.96	308.21	92.50
18℃, 24hrs dispersion	2925914.64	353.29	101.29
No dispersion STDEV.	236309.47	29.68	4.42
18℃, 24hrs dispersion STDEV.	178951.63	18.94	16.48

Table S17. Complex modulus(G*) and tack of previous and new processes in the 1/2 factory scale (n=3).

Process	Complex modulus (G*)	Tack
No dispersion	321.79	0.4172
18℃, 24hrs dispersion	367.53	0.5071
No dispersion STDEV.	30.01	0.0215
18℃, 24hrs dispersion STDEV.	25.11	0.0043

Table S18. MoD(%) of previous and new processes in the 1/2 factory scale.

Process	MoD(%)
No dispersion	3.16
18℃, 24hrs dispersion	3.43

Table S19. Complex viscosity, storage modulus(G′) and loss modulus(G′′) of previous (n=1) and new (n=3) processes in the factory scale.

Process	Complex viscosity(cP)	G′(Pa)	G′′(Pa)
No dispersion, NaCl puri.	1906628.69	228.72	71.36
18℃, 24hrs dispersion, PBS puri.	2641436.50	314.00	107.40
No dispersion, NaCl puri. STDEV.	-	-	-
18℃, 24hrs dispersion, PBS puri. STDEV.	285151.93	30.05	22.99

Table S20. Complex modulus(G*) and tack of previous (n=1) and new (n=3) processes in the factory scale.

Process	Complex modulus (G*)	Tack
No dispersion, NaCl puri.	239.59	0.3531
18℃, 24hrs dispersion, PBS puri.	332.02	0.4301
No dispersion, NaCl puri. STDEV.	-	-
18℃, 24hrs dispersion, PBS puri. STDEV.	35.71	0.0068

Table S21. MoD(%) of previous and new processes in the factory scale.

Process	MoD(%)
No dispersion	2.89
18℃, 24hrs dispersion	3.25
